# First detection and molecular identification of *Babesia gibsoni* and *Hepatozoon canis* in an Asiatic wild dog (*Cuon alpinus*) from Thailand

**DOI:** 10.1016/j.ijppaw.2022.02.007

**Published:** 2022-02-16

**Authors:** Benjaporn Bhusri, Paisin Lekcharoen, Tanasak Changbunjong

**Affiliations:** a The Monitoring and Surveillance Center for Zoonotic Diseases in Wildlife and Exotic Animals, Faculty of Veterinary Science, Mahidol University, Nakhon Pathom, 73170, Thailand; b Department of Pre-Clinic and Applied Animal Science, Faculty of Veterinary Science, Mahidol University, Nakhon Pathom, 73170, Thailand; c Department of Veterinary Public Health, Faculty of Veterinary Science, Chulalongkorn University, Bangkok, 10130, Thailand

**Keywords:** Asiatic wild dog, *Babesia*, Dhole, *Hepatozoon*, Ribosomal RNA, Tick

## Abstract

*Babesia* spp. and *Hepatozoon* spp. are apicomplexan parasites that infect a wide range of domestic and wild animals. The life cycle of these parasites requires a tick vector as a definitive host and various vertebrates as reservoir hosts. The objective of this study was to detect and characterize *Babesia* spp. and *Hepatozoon* spp. in an Asiatic wild dog (*Cuon alpinus*) from a natural habitat in northeastern Thailand. Heart and spleen samples of an adult male wild dog were screened for *Babesia* spp. and *Hepatozoon* spp. by polymerase chain reaction (PCR) of the partial fragment of 18S ribosomal RNA gene sequence. Both *Babesia* sp. and *Hepatozoon* sp. were detected in the spleen of the wild dog. Nucleotide sequence and phylogenetic analyses showed that the detected parasites were *Babesia gibsoni* and *Hepatozoon canis*. This is the first report of *B. gibsoni* and *H. canis* in the Asiatic wild dog from Thailand using PCR. Our results indicate that wild dogs can serve as a potential reservoir of the protozoan parasites and that they may play an important role in the transmission of these parasites to other wild or domestic canids.

## Highlights

• The Asiatic wild dog (*Cuon alpinus*) is involved in the cycle of tick-borne pathogens (TBP).
• This is the first report of *Babesia gibsoni* and *Hepatozoon canis* co-infection in an Asiatic wild dog.
• Wild dogs may be a source of TBP for other wild and domestic canids.

## Graphical abstract

**Figure:**
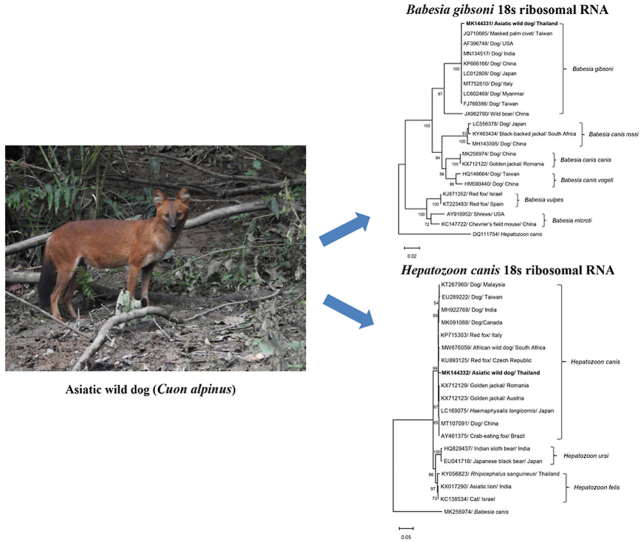

## 1 Introduction

The Asiatic wild dog or dhole (*Cuon alpinus*) is a medium-sized mammal in the family Canidae occurring in southern and southeastern Asia. It is a social, pack-living species that prefers to hunt medium to large-sized ungulate prey (Jenks et al., 2014). This species is mainly found in several protected areas in Thailand but can sometimes be found outside parks or sanctuaries (Jenks et al., 2012). Owing to a decline in its population from habitat loss, hunting, and invasion by domestic animals and their related pathogens, *C. alpinus* has been classified as an endangered species (Kamler et al., 2015).

Protozoan parasites of the genera *Babesia* and *Hepatozoon* are tick-borne pathogens that infect a wide variety of mammals, birds, reptiles and amphibians (Smith, 1996; Baneth et al., 2003; Peirce and Parsons, 2012; Schnittger et al., 2012). These parasites are associated with negative worldwide economic, clinical and epidemiological impacts, mainly in farm and pet animals (Smith, 1996; Schnittger et al., 2012). *Babesia* spp., which cause babesiosis, are transmitted by ixodid ticks. The clinical signs of babesiosis are mainly hemolysis and anemia (Margalit Levi et al., 2018). The severity of infection by *Babesia* spp. can range from subclinical or chronic to severe or fatal, depending on the virulence of the species and the immune response of the hosts (Decaro et al., 2013; Alvarado-Rybak et al., 2016; Solano-Gallego et al., 2016). *Hepatozoon* spp., which cause hepatozoonosis, are transmitted by ingestion of an infected tick. The infection by *Hepatozoon* spp. often presents subclinical infection, but can occasionally present clinical signs such as weight loss, lethargy, fever, anemia and lymphadenopathy (Baneth and Weigler, 1997; Baneth et al., 2003; Alvarado-Rybak et al., 2016). The same species of *Babesia* and *Hepatozoon* have been reported in both domestic dogs and wild carnivores. From an evolutionary perspective, the parasites can be transferred from wild canines to domestic dogs living in the same geographical areas. In addition, the transmission of the parasitic diseases between wild and domestic canines also depends on other factors such as the canine host species-specificity, the different habitats of domestic and wild canines, and the exposure to a different spectrum of tick-borne vectors (Baneth, 2001; Penzhorn, 2011; Hodžić et al., 2018; Margalit Levi et al., 2018).

In Thailand, *Babesia* and *Hepatozoon* infections have been reported in domestic and wild animals and also in tick vectors (Jittapalapong et al., 2006; Salakij et al., 2008, 2010; Laummaunwai et al., 2014; Piratae et al., 2015; Sumrandee et al., 2015; Bhusri et al., 2017). However, there are not previous records of *Babesia* and *Hepatozoon* infections in the Asiatic wild dog. Therefore, the objective of this study was to detect and characterize *Babesia* spp. and *Hepatozoon* spp. in the Asiatic wild dog from a natural habitat in northeastern Thailand using the partial 18S ribosomal RNA (18S rRNA) gene sequence.

## 2 Materials and methods

### 2.1 Sample collection

In August 2018, heart and spleen samples of an Asiatic wild dog from a natural habitat in northeastern Thailand were collected and sent to the Monitoring and Surveillance Center for Zoonotic Diseases in Wildlife and Exotic Animals (MoZWE), Faculty of Veterinary Science, Mahidol University to screen for parasitic infections. The heart and spleen samples were collected by wildlife authorities from a wild dog found dead in a forested area. Our study protocol was approved by the Faculty of Veterinary Science, Mahidol University Animal Care and Use Committee (Ref. MUVS-2019-02-07).

### 2.2 DNA extraction and 18S rRNA amplification

Genomic DNA was extracted from the heart and spleen samples using a DNeasy Blood & Tissue Kit (QIAGEN, Germany) according to the manufacturer's protocol. The polymerase chain reaction (PCR) assays were run to detect *Babesia* spp. and *Hepatozoon* spp. infections. A partial fragment of 18S rRNA gene was amplified using multiplex-PCR. For *Babesia* spp., a 619 base pair (bp) fragment was amplified by a primer pair consisting of a forward primer Ba-103F (5′-CCAATCCTGACACAGGGAGGTAGTGACA-3′) and a reverse primer Ba-721R (5′-CCCCAGAACCCAAAGACTTTGATTTCTCTCAAG-3′), whereas *Hepatozoon* spp, a 737 bp fragment was amplified by a primer pair consisting of a forward primer Hep-001F (5′-CCTGGCTATACATGAGCAAAATCTCAACTT-3′) and a reverse primer Hep-737R (5′-CCAACTGTCCCTATCAATCATTAAAGC-3′) (Kledmanee et al., 2009). The multiplex-PCR reaction mixture consisted of 12.5 μl of QIAGEN Multiplex-PCR Master Mix, 10.25 μl of nuclease-free water, 0.125 μl of each primer (100 μM), and 2 μl of DNA template in a total reaction volume of 25 μl. The PCR thermocycling conditions consisted of one step of 95 °C for 15 min, followed by 35 cycles of 94 °C for 45 s, 65 °C for 45 s, and 72 °C for 90 s, with a final extension step at 72 °C for 10 min. Amplified PCR products were separated by gel electrophoresis on a 2% agarose gel, visualized with GelRed™ (Biotium, USA) under UV light. The GeneRuler™ 100 bp DNA ladder (Thermo Scientific) was used to estimate the size of DNA fragments on the gel.

### 2.3 DNA cloning

The PCR products were cloned using the pGEM®-T Easy Vector System (Promega) according to the manufacturer's protocol. Briefly, each PCR product was ligated into the vector, before the ligated vector was transformed into the competent *E. coli* DH5α. The competent cells were screened for recombinant vector by blue/white selection technique in a Luria-Bertani (LB) plate supplemented with ampicillin/IPTG/X-Gal. The white colony carrying the PCR product was cultured in LB medium with the addition of an antibiotic before the plasmids (the ligation vectors) were extracted using the QIAprep Spin Miniprep Kit for the next step DNA sequencing.

### 2.4 DNA sequencing and phylogenetic analysis

DNA sequencing of PCR products was performed using an ABI 3730 XL DNA analyzer at Bioneer (Daejeon, Korea) with T7 universal primer. The nucleotide sequences were edited and analyzed using MEGA 11 (Molecular Evolutionary genetic analysis) software (Tamura et al., 2021). Multiple alignments of all nucleotide sequences were done using ClustalW within MEGA 11. The nucleotide sequence result was compared to sequences deposited in the GenBank database using the Basic Local Alignment Search Tool (BLAST) (http://blast.ncbi.nlm.nih.gov/Blast.cgi). A phylogenetic tree based on neighbor-joining (NJ) method with bootstrapping (1000 replicates) was constructed by MEGA 11 software (Tamura et al., 2021).

## 3 Results and discussion

**Fig. 1:**
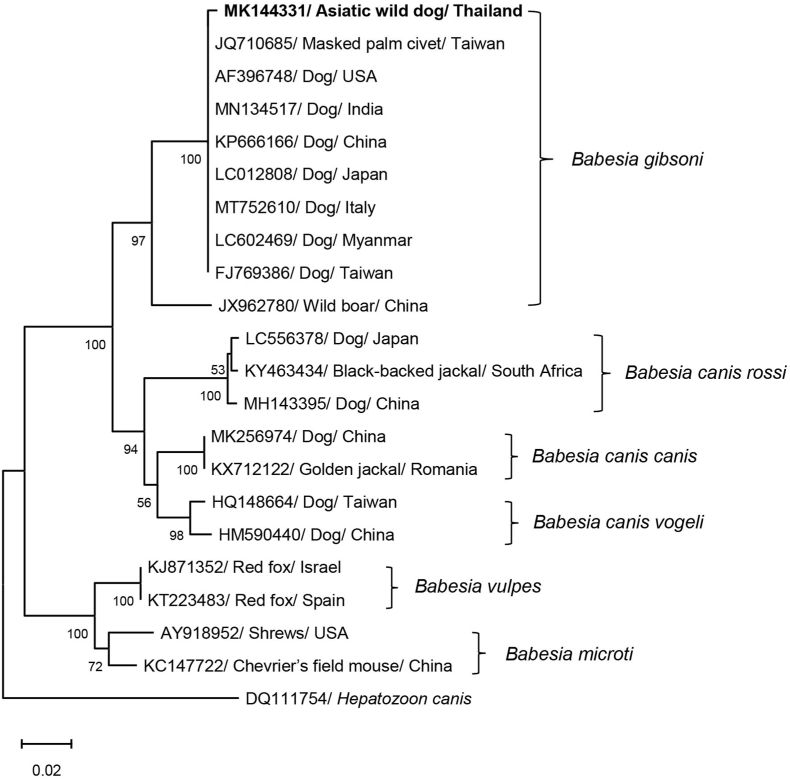
Neighbor-joining (NJ) tree of the *Babesia* partial 18S ribosomal RNA (18S rRNA) gene sequence. *Babesia gibsoni* (MK144331) was amplified from an Asiatic wild dog in Thailand and analyzed for comparison with other *Babesia* spp. from the GenBank database. The numbers on branches indicate percent bootstrap support based on 1000 bootstrap replications and only bootstrap values ≥ 50% are shown.

**Fig. 2:**
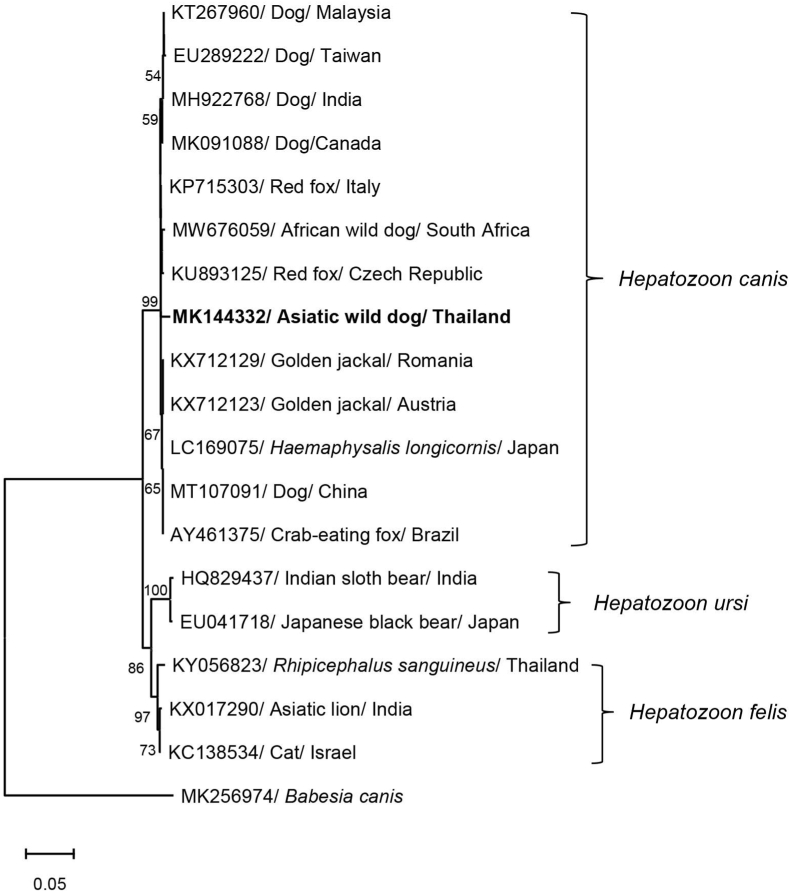
Neighbor-joining (NJ) tree of the *Hepatozoon* partial 18S ribosomal RNA (18S rRNA) gene sequence. *Hepatozoon canis* (MK144332) was amplified from an Asiatic wild dog in Thailand and analyzed for comparison with other *Hepatozoon* spp. from the GenBank database. The numbers on branches indicate percent bootstrap support based on 1000 bootstrap replications and only bootstrap values ≥ 50% are shown.

Both *Babesia* sp. and *Hepatozoon* sp. were detected in the spleen of the adult male wild dog. The nucleotide sequences of both parasites were compared with available sequences in the GenBank database by BLAST. The sequence of *Babesia* sp. showed 99.64% identity to *B. gibsoni* sequences (GenBank: MN134517), whereas the nucleotide sequence of *Hepatozoon* sp. showed 98.51% identity to *H. canis* (GenBank: MN393911). The sequences of *B. gibsoni* (GenBank: MK144331) and *H. canis* (GenBank: MK144332) were used in the phylogenetic analysis. The phylogenetic tree of the 18S rRNA of *B. gibsoni* and *H. canis* and other related species was established using an NJ method (Fig. 1, Fig. 2). *Babesia gibsoni* isolated from the wild dog in this study was included in the cluster with *B. gibsoni* isolated from the domestic dog and other wild animals; i.e. masked palm civet in Taiwan and wild boar in China. The *B. gibsoni* sequences were clearly separated from other *Babesia* spp. including *B. canis*, *B. microti* and *B. vulpes*. *Hepatozoon canis* isolated from wild dog in this study was clustered with other *H. canis* isolated from domestic dog and many wild animals; i.e. African wild dog in Africa, red fox in Czech Republic and Italy, and golden jackal in Austria and Romania. The *H. canis* sequences were clearly separated from other *Hepatozoon* spp. including *H. felis* and *H. ursi*.

This study reports for the first time the occurrence of *B. gibsoni* and *H. canis* in the Asiatic wild dog. The post-mortem examination by veterinarians revealed that the wild dog was killed by another wild dog in a fight. Although blood parasite infections in many wildlife species are typically asymptomatic, the hosts can develop clinical signs under certain circumstances such as unnatural hosts, stress, habitat degradation, climate fluctuation, or immunosuppression (Williams et al., 2014). In such cases, a wild dog may present subclinical or mild infections according to the clinical manifestation of *Babesia* and *Hepatozoon* infections in wild canids which are typically subclinical (McCully et al., 1975; Schoeman, 2009; Penzhorn, 2011). *Babesia* infection can cause severe illness, especially in young and immunosuppressed dogs. Colly and Nesbit (1992) reported a fatal acute babesiosis in a juvenile African wild dog (*Lycaon pictus*). Additionally, the stress in captive wild animals can enhance the severity of babesiosis, since in many cases infected wild animals died soon after capture or translocation (Penzhorn, 2006).

Canine babesiosis, caused by *B. canis vogeli*, is one of the most important tick-borne diseases of dogs in Thailand (Niwetpathomwat et al., 2006; Piratae et al., 2015; Juasook et al., 2016). However, there is no previous report of canine babesiosis in wild canids in the country. In this study, the nucleotide sequence of *Babesia* sp, was highly similar to that of *B. gibsoni* corresponding with the result from phylogenetic analysis. The infection by *B. gibsoni* has been found worldwide, especially from domestic dogs in Asian countries (Groves and Yap, 1968; He et al., 2017; Do et al., 2021). Furthermore, *B. gibsoni* has also been reported in wild carnivores such as golden jackal and red fox (Penzhorn, 2006).

Canine hepatozoonosis is also an important tick-borne disease of dogs in Thailand (Jittapalapong et al., 2006). In this study, the nucleotide sequence of *Hepatozoon* sp. from the wild dog was highly similar to that of *H. canis*. The result from the phylogenetic analysis revealed that *H. canis* (MK144332) was included in the group with other isolated *H. canis.* The infection by *H. canis* has been reported in domestic dogs from many countries of Asia, Europe, Africa, and America (Oyamada et al., 2005; Allen et al., 2011; Cimpan et al., 2020; Guo et al., 2020). Additionally, infection with *H. canis* have been reported in many wild carnivores such as African wild dog (Netherlands et al., 2021), crab-eating fox (Criado-Fornelio et al., 2006), golden jackal (Farkas et al., 2014), and red fox (Farkas et al., 2014; Imre et al., 2015; Ebani et al., 2017).

The transmission of *B. gibsoni* and *H. canis* is usually attributed to hard tick species. *Rhipicephalus sanguineus*, the brown dog tick (spread worldwide with domestic dogs) is the main vector of *H. canis* (Baneth, 2001) and *B. canis vogeli* (Penzhorn, 2011), but *B. gibsoni* in Asia is mainly transmitted by the tick *Haemaphysalis longicornis* (Solano-Gallego et al., 2016). Two tick species (*Haemaphysalis lagrangei* and *H. shimoga*) and one tick genus (*Amblyomma*) were recorded from Asiatic wild dogs in Thailand (Unpublished data). These ticks might be playing an important role in transmitting those parasites in the forest. In Japan, *B. gibsoni* was detected in *Amblyomma testudinarium* collected from wild boar, while *H. canis* was detected in *H. longicornis* collected from a dog (Masatani et al., 2017). The source of both parasites in the wild dog and the responsible tick species could not be established with certainty in this study.

Based on their genetic relationship, it is important to determine the transmission pathway between domestic and wild dogs because such transmission can be devastating to endangered species as reported elsewhere (Woodroffe et al., 2012).

The co-infection by *B. canis* and *H. canis* in domestic dogs has been reported in several countries such as Costa Rica (Rojas et al., 2014), Philippines (Adao et al., 2017) and Thailand (Piratae et al., 2015; Do et al., 2021). However, a few reports of co-infection by *B. gibsoni* and *H. canis* are available. Therefore, the molecular detection of *B. gibsoni* and *H. canis* co-infection in a free-ranging Asiatic wild dog in this study is very important as it provides complementary information for monitoring disease outbreaks in other wild and domestic canids, and also for preventing wildlife extinction. To provide more efficient recommendations on the management plan for this vulnerable species, it is important that wildlife authorities should understand the extent of infection, distribution and dynamics of these parasites and their vectors in wildlife-domestic animals interface areas. The control measures focusing on prevention of wild and domestic animal interactions including restriction of domestic animal access to the protected area may reduce the risk of cross-species transmission of these parasites as well as other pathogens. Further epidemiological studies are required to clarify the relationships among parasites, ticks and reservoir hosts. More transdisciplinary engagements between conservation biologists, wildlife veterinarians and epidemiologists are important for establishing the comprehensive management plans aimed at endangered wild canid conservation.

## Credit author statement

**Benjaporn Bhusri:** Investigation, Methodology, Writing-original draft. **Paisin Lekcharoen:** Investigation, Writing-review & editing. **Tanasak Changbunjong:** Conceptualization, Investigation, Methodology, Formal analysis, Writing-review & editing.

## Declaration of competing interest

The authors declare that they have no known competing financial interests or personal relationships that could have appeared to influence the work reported in this paper.

## References

[bib1] Adao D.E.V., Herrera C.M.T., Galarion L.H., Bolo N.R., Carlos R.S., Carlos E.T., Carlos S.S., Rivera W.L. (2017). Detection and molecular characterization of *Hepatozoon canis, Babesia vogeli, Ehrlichia canis*, and *Anaplasma platys* in dogs from Metro Manila, Philippines. Korean J. Vet. Res..

[bib2] Allen K.E., Johnson E.M., Little S.E. (2011). *Hepatozoon* spp infections in the United States. Vet. Clin. North Am. Small Anim. Pract..

[bib3] Alvarado-Rybak M., Solano-Gallego L., Millán J. (2016). A review of piroplasmid infections in wild carnivores worldwide: importance for domestic animal health and wildlife conservation. Parasites Vectors.

[bib4] Baneth G., Mathew J.S., Shkap V., Macintire D.K., Barta J.R., Ewing S.A. (2003). Canine hepatozoonosis: two disease syndromes caused by separate *Hepatozoon* spp. Trends Parasitol..

[bib5] Baneth G., Weigler B. (1997). Retrospective case-control study of hepatozoonosis in dogs in Israel. J. Vet. Intern. Med..

[bib6] Baneth G. (2001). Perspectives on canine and feline hepatozoonosis. Vet. Parasitol..

[bib7] Bhusri B., Sariya L., Mongkolphan C., Suksai P., Kaewchot S., Changbunjong T. (2017). Molecular characterization of *Hepatozoon felis* in *Rhipicephalus sanguineus* ticks infested on captive lions (*Panthera leo*). J. Parasit. Dis..

[bib8] Cimpan A.A., Nachum-Biala Y., Ben-Shitrit B., Miron L., Baneth G. (2020). Epidemiological study of canine babesiosis and hepatozoonosis in the south of Romania. Acta Parasitol..

[bib9] Colly L.P., Nesbit J.W. (1992). Fatal acute babesiosis in a juvenile wild dog (*Lycaon pictus*). J. S. Afr. Vet. Assoc..

[bib10] Criado-Fornelio A., Ruas J.L., Casado N., Farias N.A., Soares M.P., Müller G., Brumt J.G., Berne M.E., Buling-Saraña A., Barba-Carretero J.C. (2006). New molecular data on mammalian *Hepatozoon* species (Apicomplexa: adeleorina) from Brazil and Spain. J. Parasitol..

[bib11] Decaro N., Larocca V., Parisi A., Losurdo M., Lia R.P., Greco M.F., Miccolis A., Ventrella G., Otranto D., Buonavoglia C. (2013). Clinical bovine piroplasmosis caused by *Babesia occultans* in Italy. J. Clin. Microbiol..

[bib12] Do T., Ngasaman R., Saechan V., Pitaksakulrat O., Liu M., Xuan X., Inpankaew T. (2021). First molecular detection of *Babesia gibsoni* in stray dogs from Thailand. Pathogens.

[bib13] Ebani V.V., Rocchigiani G., Nardoni S., Bertelloni F., Vasta V., Papini R.A., Verin R., Poli A., Mancianti F. (2017). Molecular detection of tick-borne pathogens in wild red foxes (*Vulpes vulpes*) from Central Italy. Acta Trop..

[bib14] Farkas R., Solymosi N., Takács N., Hornyák Á., Hornok S., Nachum-Biala Y., Baneth G. (2014). First molecular evidence of *Hepatozoon canis* infection in red foxes and golden jackals from Hungary. Parasites Vectors.

[bib15] Groves M.G., Yap L.F. (1968). Babesia gibsoni in a dog. J. Am. Vet. Med. Assoc..

[bib16] Guo W.P., Xie G.C., Xue Z.Q., Yu J.J., Jian R., Du L.Y., Li Y.N. (2020). Molecular detection of *Hepatozoon canis* in dogs and ticks in Shaanxi province, China. Comp. Immunol. Microbiol. Infect. Dis. 2020.

[bib17] He L., Miao X., Hu J., Huang Y., He P., He J., Yu L., Malobi N., Shi L., Zhao J. (2017). First molecular detection of *Babesia gibsoni* in dogs from Wuhan, China. Front. Microbiol..

[bib18] Hodžić A., Mrowietz N., Cézanne R., Bruckschwaiger P., Punz S., Habler V.E., Tomsik V., Lazar J., Duscher G.G., Glawischnig W., Fuehrer H.P. (2018). Occurrence and diversity of arthropod-transmitted pathogens in red foxes (Vulpes vulpes) in western Austria, and possible vertical (transplacental) transmission of *Hepatozoon canis*. Parasitology.

[bib19] Imre M., Dudu A., Ilie M.S., Morariu S., Imre K., Dărăbuş G. (2015). Molecular survey of *Hepatozoon canis* in red foxes (*Vulpes vulpes*) from Romania. J. Parasitol..

[bib20] Jenks K.E., Kitamura S., Lynam A.J., Ngoprasert D., Chutipong W., Steinmetz R., Sukmasuang R., Grassman L.I., Cutter P., Tantipisanuh N., Bhumpakphan N., Gale G.A., Reed D.H., Leimgruber P., Songsasen N. (2012). Mapping the distribution of dholes, *Cuon alpinus* (Canidae, carnivora), in Thailand. Mammalia.

[bib21] Jenks K.E., Songsasen N., Kanchanasaka B., Leimgruber P., Fuller T.K. (2014). Local people's attitudes and perceptions of dholes (*Cuon alpinus*) around protected areas in southeastern Thailand. Trop. Conserv. Sci..

[bib22] Jittapalapong S., Rungphisutthipongse O., Maruyama S., Schaefer J.J., Stich R.W. (2006). Detection of *Hepatozoon canis* in stray dogs and cats in Bangkok, Thailand. Ann. N. Y. Acad. Sci..

[bib23] Juasook A., Boonmars T., Sriraj P., Aukkanimart R., Sudsan P., Wonkchalee N., Boonjaraspinyo S., Laummaunwai P., Maleewong W., Ployngam T., Jitasombuti P., Ratanasuwan P. (2016). Misdiagnose tick-borne pathogens in domestic dogs in Khon Kaen province, demonstrated using molecular identification. Chiang Mai Vet. J..

[bib24] Kamler J.F., Songsasen N., Jenks K., Srivathsa A., Sheng L., Kunkel K. (2015). Cuon alpinus. The IUCN Red List of Threatened Species 2015: e.T5953A72477893.

[bib25] Kledmanee K., Suwanpakdee S., Krajangwong S., Chatsiriwech J., Suksai P., Suwannachat P., Sariya L., Buddhirongawatr R., Charoonrut P., Chaichoun K. (2009). Development of multiplex polymerase chain reaction for detection of *Ehrlichia canis, Babesia* spp. and *Hepatozoon canis* in canine blood. Southeast Asian J. Trop. Med. Publ. Health.

[bib26] Laummaunwai P., Sriraj P., Aukkanimart R., Boonmars T., Boonjaraspinyo S., Sangmaneedet S., Potchimplee P., Khianman P., Maleewong W. (2014). Molecular detection and treatment of tick-borne pathogens in domestic dogs in Khon Kaen, northeastern Thailand. Southeast Asian J. Trop. Med. Publ. Health.

[bib27] Margalit Levi M., Nachum-Biala Y., King R., Baneth G. (2018). A survey of *Babesia* spp. and *Hepatozoon* spp. in wild canids in Israel. Parasites Vectors.

[bib28] Masatani T., Hayashi K., Andoh M., Tateno M., Endo Y., Asada M., Kusakisako K., Tanaka T., Gokuden M., Hozumi N., Nakadohzono F., Matsuo T. (2017). Detection and molecular characterization of *Babesia, Theileria*, and *Hepatozoon* species in hard ticks collected from Kagoshima, the southern region in Japan. Ticks Tick Borne Dis.

[bib29] McCully R.M., Basson P.A., Bigalke R.D., De Vos V., Young E. (1975). Observations on naturally acquired hepatozoonosis of wild carnivores and dogs in the Republic of South Africa. Onderstepoort J. Vet. Res..

[bib30] Netherlands E.C., Stroebel C., du Preez L.H., Shabangu N., Matjila P.T., van Schalkwyk O.L., Penzhorn B.L. (2021). Molecular confirmation of high prevalence of species of Hepatozoon infection in free-ranging African wild dogs (*Lycaon pictus*) in the Kruger National Park, South Africa. Int. J. Parasitol. Parasites Wildl..

[bib31] Niwetpathomwat A., Somporn L., Suvarnavibhaja S., Assarasakorn S. (2006). A retrospective study of clinical hematology and biochemistry of canine babesiosis on hospital populations in Bangkok, Thailand. Comp. Clin. Pathol..

[bib32] Oyamada M., Davoust B., Boni M., Dereure J., Bucheton B., Hammad A., Itamoto K., Okuda M., Inokuma H. (2005). Detection of *Babesia canis rossi, B. canis vogeli*, and *Hepatozoon canis* in dogs in a village of eastern Sudan by using a screening PCR and sequencing methodologies. Clin. Diagn. Lab. Immunol..

[bib33] Peirce M.A., Parsons N.J. (2012). Babesia ugwidiensis, a new species of Avian piroplasm from Phalacrocoracidae in South Africa. Parasite.

[bib34] Penzhorn B.L. (2006). Babesiosis of wild carnivores and ungulates. Vet. Parasitol..

[bib35] Penzhorn B.L. (2011). Why is southern African canine babesiosis so virulent? An evolutionary perspective. Parasites Vectors.

[bib36] Piratae S., Pimpjong K., Vaisusuk K., Chatan W. (2015). Molecular detection of *Ehrlichia canis, Hepatozoon canis* and *Babesia canis vogeli* in stray dogs in Mahasarakham province, Thailand. Ann. Parasitol.

[bib37] Rojas A., Rojas D., Montenegro V., Gutiérrez R., Yasur-Landau D., Baneth G. (2014). Vector-borne pathogens in dogs from Costa Rica: first molecular description of *Babesia vogeli* and *Hepatozoon canis* infections with a high prevalence of monocytic ehrlichiosis and the manifestations of co-infection. Vet. Parasitol..

[bib38] Salakij C., Salakij J., Narkkong N.A., Sirinarumitr T., Pattanarangsan R. (2008). Hematologic, cytochemical, ultrastructural, and molecular findings of Hepatozoon-infected flat-headed cats (*Prionailurus planiceps*). Vet. Clin. Pathol..

[bib39] Salakij C., Sirinarumitr T., Tongthainun D. (2010). Molecular characterization of *Hepatozoon* species in a leopard cat (*Prionailurus bengalensis*) from Thailand. Vet. Clin. Pathol..

[bib40] Schnittger L., Rodriguez A.E., Florin-Christensen M., Morrison D.A. (2012). Babesia: a world emerging. Infect. Genet. Evol..

[bib41] Schoeman J.P. (2009). Canine babesiosis. Onderstepoort J. Vet. Res..

[bib42] Smith T.G. (1996). The genus *Hepatozoon* (apicomplexa: adeleina). J. Parasitol..

[bib43] Solano-Gallego L., Sainz A., Roura X., Estrada-Peña A., Miró G. (2016). A review of canine babesiosis: the European perspective. Parasites Vectors.

[bib44] Sumrandee C., Baimai V., Trinachartvanit W., Ahantarig A. (2015). *Hepatozoon* and *Theileria* species detected in ticks collected from mammals and snakes in Thailand. Ticks Tick Borne Dis.

[bib45] Tamura K., Stecher G., Kumar S. (2021). MEGA11: molecular evolutionary genetics analysis. Mol. Biol. Evol..

[bib46] Williams B.M., Berentsen A., Shock B.C., Teixiera M., Dunbar M.R., Becker M.S., Yabsley M.J. (2014). Prevalence and diversity of Babesia, *Hepatozoon, ehrlichia*, and *bartonella* in wild and domestic carnivores from Zambia, Africa. Parasitol. Res..

[bib47] Woodroffe R., Prager K.C., Munson L., Conrad P.A., Dubovi E.J., Mazet J.A. (2012). Contact with domestic dogs increases pathogen exposure in endangered African wild dogs (*Lycaon pictus*). PLoS One.

